# Impaired Immune Response to Primary but Not to Booster Vaccination Against Hepatitis B in Older Adults

**DOI:** 10.3389/fimmu.2018.01035

**Published:** 2018-05-15

**Authors:** Birgit Weinberger, Mariëlle C. Haks, Roelof A. de Paus, Tom H. M. Ottenhoff, Tanja Bauer, Beatrix Grubeck-Loebenstein

**Affiliations:** ^1^Institute for Biomedical Aging Research, Universität Innsbruck, Innsbruck, Austria; ^2^Department of Infectious Diseases, Leiden University Medical Center, Leiden, Netherlands; ^3^Institute of Virology, Technische Universität München/Helmholtz Zentrum München, Munich, Germany

**Keywords:** hepatitis B virus, vaccine, primary vaccination, booster vaccination, elderly, gene expression profiling

## Abstract

Many current vaccines are less immunogenic and less effective in elderly compared to younger adults due to age-related changes of the immune system. Most vaccines utilized in the elderly contain antigens, which the target population has had previous contact with due to previous vaccination or infection. Therefore, most studies investigating vaccine-induced immune responses in the elderly do not analyze responses to neo-antigens but rather booster responses. However, age-related differences in the immune response could differentially affect primary versus recall responses. We therefore investigated the impact of age on primary and recall antibody responses following hepatitis B vaccination in young and older adults. Focused gene expression profiling was performed before and 1 day after the vaccination in order to identify gene signatures predicting antibody responses. Young (20–40 years; *n* = 24) and elderly (>60 years; *n* = 17) healthy volunteers received either a primary series (no prior vaccination) or a single booster shot (documented primary vaccination more than 10 years ago). Antibody titers were determined at days 0, 7, and 28, as well as 6 months after the vaccination. After primary vaccination, antibody responses were lower and delayed in the elderly compared to young adults. Non-responders after the three-dose primary series were only observed in the elderly group. Maximum antibody concentrations after booster vaccination were similar in both age groups. Focused gene expression profiling identified 29 transcripts that correlated with age at baseline and clustered in a network centered around type I interferons and pro-inflammatory cytokines. In addition, smaller 8- and 6-gene signatures were identified at baseline that associated with vaccine responsiveness during primary and booster vaccination, respectively. When evaluating the kinetic changes in gene expression profiles before and after primary vaccination, a 33-gene signature, dominated by IFN-signaling, pro-inflammatory cytokines, inflammasome components, and immune cell subset markers, was uncovered that was associated with vaccine responsiveness. By contrast, no such transcripts were identified during booster vaccination. Our results document that primary differs from booster vaccination in old age, in regard to antibody responses as well as at the level of gene signatures.

**Clinical Trial Registration:**

www.clinicaltrialsregister.eu, this trial was registered at the EU Clinical Trial Register (EU-CTR) with the EUDRACT-Nr. 2013-002589-38.

## Introduction

Life expectancy is increasing worldwide, and the number of persons older than 60 years of age is expected to double, reaching 2.1 billion by 2050 (1). The incidence and severity of many infectious diseases is high in the elderly compared to that in younger adults (2). Vaccination is one of the most effective measures to prevent infections, but most current vaccines are less immunogenic and less efficient in older adults. Age-related changes of the immune system include a decline of naïve T and B cells (3, 4), which potentially hampers immune responses to neo-antigens. It has been shown that primary immune responses to vaccines against tick-borne encephalitis (5), Japanese encephalitis (6), hepatitis A (7), and pandemic influenza strains (8) are lower in the elderly. Reduced immunogenicity in old age has also been shown for booster vaccinations against tetanus, diphtheria (9,  10), and tick-borne encephalitis (11). However, the molecular mechanisms underlying age-related hyporesponsiveness to vaccination remain unclear. Genome-wide RNA expression profiling has identified a clear association between chronological age and progressive changes in the transcriptional landscape of peripheral blood cells. Significant age-related changes were found in the transcript levels of markers involved in, e.g., immunosenescence, inflammation, and oxidative stress (12). Moreover, many of the identified genes were highly and preferentially expressed in naïve and memory T and B cells and may thus reflect age-related changes in immune function (13, 14). Hence, pre-immunization transcriptomic profiles and/or changes in gene expression patterns in blood, resulting from the elicited innate and adaptive immune responses after vaccination, could potentially be used as biomarkers to classify and predict vaccine responsiveness and be key to a better understanding of (hypo)responsiveness to vaccination in the elderly population.

Immune responses after influenza and pneumococcal vaccination—the most studied vaccines in the elderly—are usually a mixture of primary and recall responses, as natural contact with various influenza strains and pneumococcal serotypes is frequent, but vaccines might also contain neo-antigens. It is therefore rare that primary responses and purely vaccine-induced recall responses (without natural exposure) to the same antigens are investigated in the elderly. We have chosen hepatitis B virus surface antigen (HBsAg) as a model antigen since natural exposure to hepatitis B virus (HBV) is relatively rare in Austria (15), and primary as well as booster vaccinations can be performed in young and older adults following national recommendations.

In addition to the value of HBsAg as a model antigen, HBV is also of clinical relevance for the older population. Acute infection with HBV is mainly recognized in young adults with high-risk behaviors, but is also relevant in old age (16). Older adults with viral hepatitis have a higher mortality rate than younger patients, which can be partially explained by underlying comorbidities, but also by a diminished immune response, metabolic and nutritional deficiencies, and age-related anatomic changes of the liver (17–19). Progression of acute HBV infection to chronicity occurs in less than 5% of young adults, but is observed more frequently in the elderly. In an outbreak in a nursing home in Japan, almost 60% of the infected elderly became HBsAg carriers (20). The prevalence of HBsAg (>6 months), which indicates chronic infection, is higher in nursing home residents compared with non-institutionalized populations, and the risk of transmission is higher in these facilities (21).

Childhood vaccination against hepatitis B is recommended in many countries. Most European countries and the USA also recommend primary vaccination of adults and booster vaccinations for persons with an increased risk for infection. In addition to persons with high-risk lifestyles (use of injected drugs, high-risk sexual behavior) or occupational risk factors (e.g., health-care personnel, police and other emergency service personnel, persons working with refugees), these risk groups include household contacts of chronically infected patients, patients with chronic liver disease, persons undergoing dialysis or regularly receiving plasma products, and persons under immunosuppressive therapy. All of these risk groups are also composed of older persons (22–27). In addition, vaccination is recommended for persons traveling to areas with a high prevalence of chronic HBV infection. Due to increased life expectancy as well as improved health status and mobility of elderly persons, the number of older long-distance travelers rises. The exact data on the extent of travel by older adults are limited, but data from several countries indicate that a substantial fraction of long-distance travelers are over the age of 65 (28, 29). Primary immunization against hepatitis B is performed relatively late in life, e.g., in travelers, household contacts of chronic hepatitis B carriers, or persons at risk in long-term care facilities. For this reason, the goal of this study was to compare the outcome of primary versus booster vaccination against hepatitis B in young and elderly persons from the same geographical and social background at the level of antibody response and gene signatures.

## Materials and Methods

### Study Cohort

Two study cohorts of young and old healthy adults were recruited. Group A had never been vaccinated against hepatitis B and received a primary immunization series (three doses, 0–1–6 months). Serum was collected at days 0, 7, and 28 after each vaccination as well as 6 months after the last dose. Group B comprised persons who had received a full primary vaccination against hepatitis B at least 10 years ago and now received a single booster vaccination. Serum was collected at days 0, 7 and 28, and 6 months after the vaccination. Whole blood was collected in PAXgene blood RNA tubes (PreAnalytiX GmbH, Switzerland) before and 1 day after the first vaccination for expression analysis. Each group included young and old donors (Table 1). Persons with chronic viral infection (human immunodeficiency virus, hepatitis B virus, hepatitis C virus), transplant recipients, and patients under immunosuppressive or chemotherapy were not included in the study. Twinrix^®^ (Glaxo Smith Kline, UK), which contains 720 ELISA units of inactivated hepatitis A virus and 20-µg recombinant HBsAg adjuvanted with Al(OH)_3_ and AlPO_4_, was used for all vaccinations. The objective of this study was to measure the hepatitis B-specific immune response. The protocol was approved by the ethics committee of the Innsbruck Medical University. All participants gave their written informed consent in accordance with the Declaration of Helsinki. This trial was registered at the EU Clinical Trial Register (EU-CTR) with the EUDRACT-Nr. 2013-002589-38.

**Table 1:** Characteristics of study cohort.

		*n*	Median age (years)	Age range (years)	Cytomegalovirus seropositive
Group A	Young	12^a^	27.5	20–40	27%^c^
	Old	9^b^	72	60–84	67%

Group B	Young	12	29.5	20–38	58%
	Old	8	68.5	62–74	50%

*^a^One person was excluded from the analysis because HBsAg-specific antibodies were detectable at day 0 indicating previous contact with HBV*.

*^b^Two persons were excluded from the analysis because HBsAg-specific antibodies were detectable at day 0 indicating previous contact with HBV*.

*^c^CMV serology was not available for one person*.

### Detection of HBsAg-Specific and Cytomegalovirus (CMV)-Specific Antibodies

Serum levels of HBsAg-specific antibodies (anti-HBs) were quantified using the ARCHITECT^®^ chemiluminescence microparticle immunoassay (Abbott Diagnostics, Wiesbaden, Germany). Samples with anti-HBs levels below the limit of detection (0.9 IU/l) were set to 0.45 IU/l and samples with antibody levels above the dynamic range of the assay (10,000 IU/l) were diluted and re-tested. Serum levels of CMV-specific antibodies were quantified by ELISA using the Serion ELISA classic CMVs IgG Kit (Virion/Serion GmbH, Würzburg, Germany). Individuals with antibody concentrations below 25 PEI-U/ml are considered to be seronegative and values above 40 PEI-U/ml indicate seropositive individuals. Values between 25 and 40 PEI-U/ml are considered borderline, but were not seen in this study.

### RNA Isolation

Total RNA from venipuncture PAXgene blood collection tubes was extracted using the PAXgene Blood mRNA kit (PreAnalytiX GmbH, Switzerland) according to the manufacturer’s protocol. The RNA yield was determined by a NanoDrop ND-1000 spectrophotometer (NanoDrop Technologies, Wilmington, DE, USA), while the quality and integrity of the RNA were assessed on an Agilent 2100 BioAnalyzer (Agilent Technologies, Amstelveen, The Netherlands) using the RNA 6000 Nano Chip kit. The average RNA integrity number of the total RNA samples obtained from PAXgene tubes was 9.6 ± 0.05.

### Dual-Color Reverse-Transcription Multiplex Ligation-Dependent Probe Amplification (dcRT-MLPA)

Focused gene expression profiling using dcRT-MLPA was performed in triplicate as described in detail elsewhere (30). Briefly, for each target-specific sequence, a specific reverse transcription (RT) primer was designed located immediately downstream of the left- and right-hand half-probe target sequence. RNA was reverse transcribed using an RT-primer mix and MMLV reverse transcriptase (Promega Benelux, Leiden, The Netherlands). Transcriptase activity was inactivated by heating at 98°C for 2 min. Following RT, the left- and right-hand half-probes were hybridized to the cDNA at 60°C overnight. Annealed half-probes were ligated using ligase 65 and subsequently amplified by PCR (33 cycles of 30 s/95°C, 30 s/58°C, 60 s/72°C, followed by 1 cycle of 20 min/72°C). Primers and probes were from Sigma-Aldrich Chemie (Zwijndrecht, The Netherlands) and MLPA reagents from MRC-Holland (Amsterdam, The Netherlands). PCR amplification products were diluted 1:10 in HiDi-formamide containing 400HD ROX size standard, denatured at 95°C for 5 min, ice-cooled, and analyzed on an Applied Biosystems 3730 capillary sequencer in GeneScan mode (BaseClear, Leiden, The Netherlands).

Reverse transcription primers and half-probes were designed by Leiden University Medical Center (Department of Infectious Diseases, Leiden, The Netherlands) (30) and comprised sequences for four housekeeping genes (including GAPDH) and 144 selected genes to profile innate, adaptive, and inflammatory immune responses (Table S1 in Supplementary Material).

Trace data were analyzed using GeneMapper software 5 package (Applied Biosystems, Bleiswijk, The Netherlands). The areas of each assigned peak (in arbitrary units) were exported for further analysis in Microsoft Excel. Data were normalized to GAPDH, and signals below the threshold value for noise cutoff in GeneMapper (log2-transformed peak area of 7.64) were assigned the threshold value. Finally, the normalized data were log2-transformed for statistical analysis.

RNA expression values were visualized by principal component analysis (PCA) plots and heatmaps using the hierarchical clustering algorithms of Clustvis.^1^ String network analysis^2^ or Ingenuity Pathway Analysis of genes that were differentially expressed was performed to identify relevant signaling pathways and regulatory networks.

### Statistical Analysis

Statistical analyses of antibody concentrations for the two age groups were performed for each time point using the nonparametric Mann–Whitney *U*-test. Triplicate measurements of log2-transformed dcRT-MLPA-derived gene expression data were analyzed for statistical significance between two populations (young versus old adults or persons with ≥10,000 IU/l versus <10,000 IU/l anti-HBs) using the Mann–Whitney *U*-test. Statistical tests were two-sided, and *p*-values were adjusted for multiple testing using the Benjamini–Hochberg correction. Findings were regarded positive when meeting the criterion on the false discovery rate (FDR) of <10% and an optional factorial change (FC) of 1.25. Since relatively few samples were included in our study, leave-one-out cross-validation (LOOCV) was performed in the PCA analysis to estimate the generalization ability of the gene signatures as classifiers, and Wilks’ Lambda test was used to test for differences between groups. The chi-squared test was used to test the independence of the two categorical variables’ age and vaccine responsiveness. Significant differences were evaluated using the Fisher’s exact test. Statistical computation was performed using SPSS 23 software.

## Results

### Antibody Responses After Primary Vaccination

**Figure 1:**
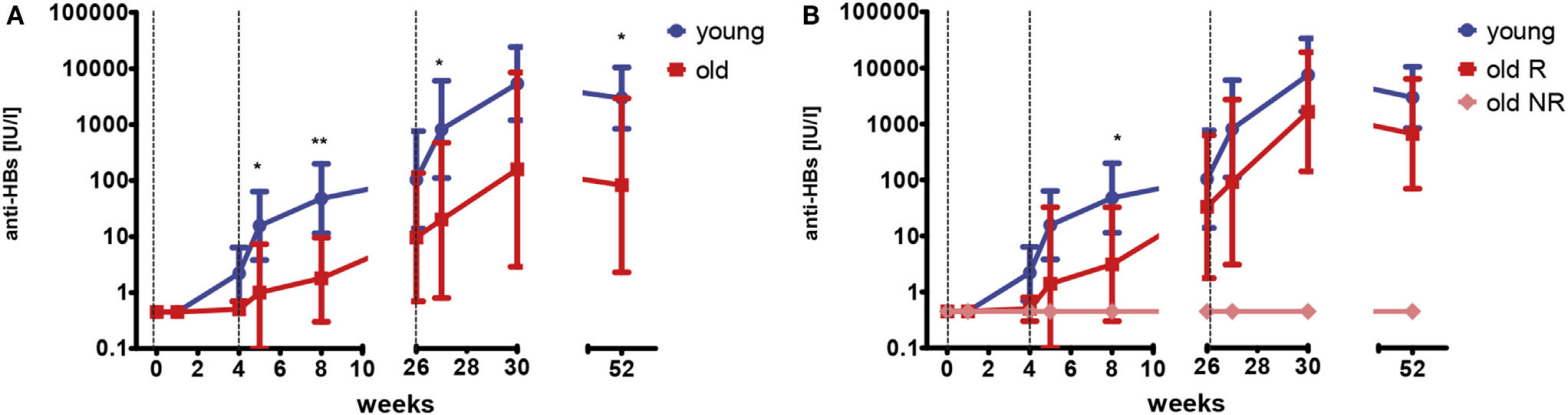
HBsAg-specific antibody concentrations after primary vaccination series. Geometric mean concentrations and 95% confidence intervals of anti-HBs are depicted. Dashed vertical lines indicate the time points of vaccination (weeks 0–4–26). The limit of detection is 0.9 IU/l. Samples below this value were set to 0.45 IU/l. **(A)** This graph depicts data for young (blue; *n* = 11) and old (red; *n* = 7) participants. Mann–Whitney *U*-test young versus old; *p < 0.05; **p < 0.01. **(B)** This graph depicts data for young (blue; *n* = 11) participants, old responders (red; *n* = 5) and old non-responders (light red; *n* = 2). Mann–Whitney *U*-test young versus old responders; *p < 0.05.

Young and old healthy adult volunteers who had never received a hepatitis A or B vaccination were recruited to receive a primary vaccination series (months 0–1–6) with a combined hepatitis A/B vaccine. HBsAg-specific antibody concentrations were measured before, 1 week, and 4 weeks after each dose, as well as 6 months after the last dose. None of the old participants mounted antibody responses after the first dose of the vaccine, whereas low levels of HBsAg-specific IgG were detectable in 40% of the young group, 4 weeks after the first dose (range 5.70–18.75 IU/l). After the second dose, antibody concentrations increased in all but one of the young participants (median 28.8-fold, range 1.8–418.8), but only in two (29%) of the older vaccines (40.3- and 68.5-fold, respectively). As expected, a further increase in antibody concentrations was observed after the third vaccination in both the young (median 31.2-fold, range 12.2–6,052.6) and the old group (median 29.3-fold, range 1–268.2). The median drop of antibody concentrations was 2.8-fold (range 0.7–9.1) within the first 6 months after the last vaccination with no age-related differences (Mann–Whitney *U*-test; *p* = 0.833). Figure 1A depicts the geometric mean antibody concentrations for both age groups.

Two of the old participants (29%) did not develop detectable antibodies (<0.9 IU/l) at any time point following primary vaccination and were classified as non-responders (NRs), whereas antibody concentrations were above 100 IU/l for all young participants. Figure 1B depicts the geometric mean antibody concentrations for the young (same as in Figure 1A), as well as the old responders (R) and old NR separately. When only the old responders were considered, their antibody concentrations were still lower than in the young group. This difference was only significant at week 8, probably due to the relatively low sample size.

### Antibody Responses After Booster Vaccination

**Figure 2:**
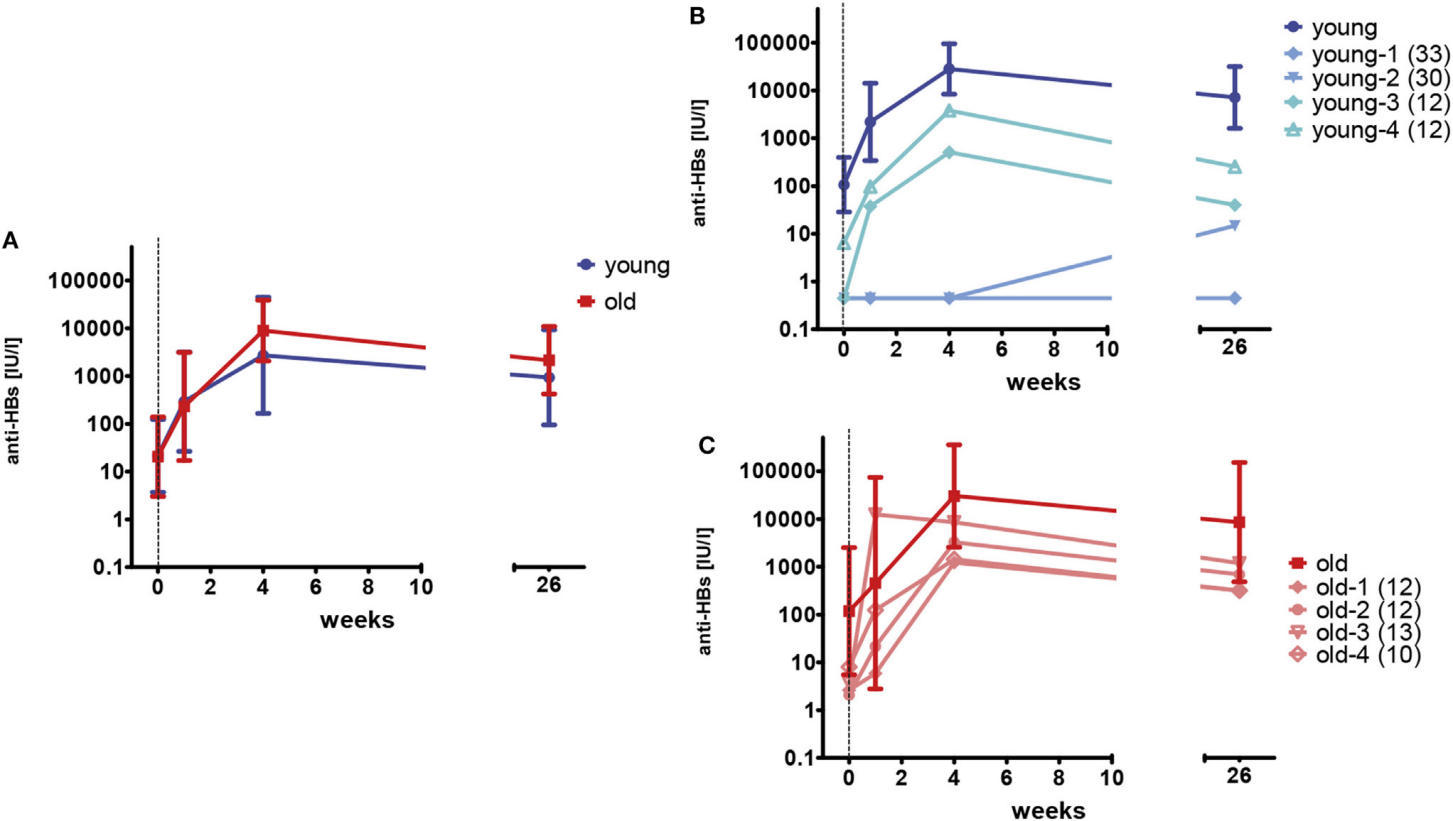
HBsAg-specific antibody concentrations after booster vaccination. One booster dose of vaccine was administered at week 0 as indicated by the vertical dashed line. The limit of detection is 0.9 IU/l. Samples below this value were set to 0.45 IU/l. **(A)** Geometric mean concentrations and 95% confidence intervals of anti-HBs are depicted for young (blue; *n* = 12) and old (red; *n* = 8) participants. **(B)** Geometric mean concentrations and 95% confidence intervals of anti-HBs are depicted for young (dark blue; *n* = 8) participants with anti-HBs concentrations of ≥10 IU/l prior to the booster vaccination. Individual antibody concentrations are shown for four young (light blue) participants, with low (<10 IU/l) anti-HBs at the time of enrollment. The numbers in brackets represent the time since the last vaccination (years) for the individual donors. **(C)** Geometric mean concentrations and 95% confidence intervals of anti-HBs are depicted for old (dark red; *n* = 4) participants with anti-HBs concentrations of ≥10 IU/l prior to the booster vaccination. Individual antibody concentrations are shown for four old (light red) participants, with low (<10 IU/l) anti-HBs at the time of enrollment. The numbers in brackets represent the time since the last vaccination (years) for the individual donors.

Young and old healthy adults, who had received a complete primary series of hepatitis vaccination more than 10 years ago, received one dose of hepatitis A/B vaccine. Anti-HBs concentrations were measured before, 1 week, 4 weeks, and 6 months after the vaccination. Antibody concentrations before the booster vaccination ranged from below the limit of detection (<0.9 IU/l) to 3270 IU/l and were similar for both age groups (young versus old, *p* = 0.910, Mann–Whitney *U*-test). Within the first 4 weeks after vaccination, anti-HBs concentrations increased in young (median 469.4-fold, range 1–1,120.5) and old (median 328.1-fold, range 1–2,325.6) participants. No age-related differences were seen at any time point (young versus old, *p* = 0.678–1.000, Mann–Whitney *U*-test). The median decline of antibodies from weeks 4 to 26 was 4.4-fold (range 1.4–14.9). Figure 2A depicts the geometric mean antibody concentrations for all participants.

Anti-HBs concentrations were below 10 IU/l in four young (33%) and four old (50%) participants at the time of enrollment. In three of the four young individuals, no anti-HBs were detectable at all. Primary vaccination had been documented for all individuals, but anti-HBs concentrations after the primary series were not available. Figures 2B,C depict the antibody concentrations for these individuals and show the geometric mean titers for young and old participants with anti-HBs concentrations of ≥10 IU/l before the booster vaccination in comparison. Two of the young individuals (young-1 and young-2) did not develop adequate antibody concentrations after the single booster shot. The primary vaccination series of these persons was performed in early childhood and therefore dated back 30 and 33 years, respectively. By contrast, the other individuals with low anti-HBs concentrations before the vaccination (young-3, young-4, and old-1–4) showed an anamnestic response to the booster dose and reached sufficient antibody concentrations. Their last hepatitis B vaccination dated back 10–13 years. A strong correlation of anti-HBs concentrations before (w0) and after the booster vaccination (w4) was observed for young (*r_S_* = 0.875; *p* < 0.001) and old (*r_S_* = 0.786; *p* = 0.021) vaccines.

### Correlation Between Pre-Immunization Transcriptomic Profiles and Vaccine Response

**Figure 3:**
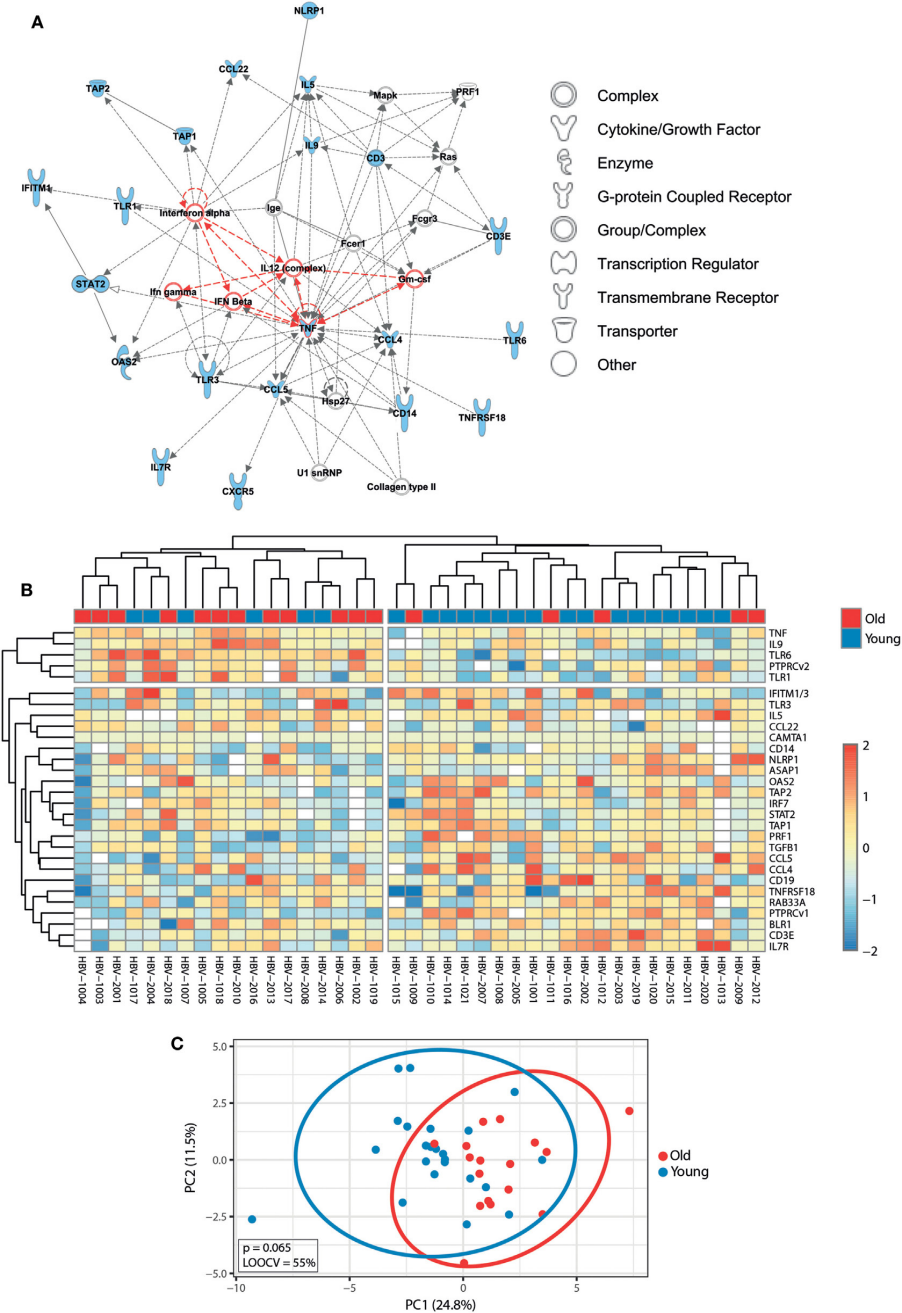
Impact of age on pre-immunization transcriptomic profiles. **(A)** Ingenuity Pathway Analysis of the 29 genes that were differentially expressed between young and old adults using a false discovery rate (FDR) of <10% (indicated in blue symbols). Type I interferons and pro-inflammatory cytokines are highlighted in red. **(B)** The median-centered gene expression of the 29-gene signature is represented using a blue to yellow to red color scale. Rows and columns correspond to the genes and the profiled samples, respectively. The age groups are presented in colored squares above each sample. **(C)** PCA analysis of the gene expression profile of the 29-gene signature. Blue and red spheres represent young and old adults, respectively.

Before exploring whether pre-immunization gene expression levels correlated with different responses to the hepatitis B vaccine between young and old adults, we first investigated the impact of age on the pre-immunization transcriptomic profiles. Mann–Whitney *U*-test was used to identify transcripts differentially expressed between young and old participants, and *p*-values were adjusted with the Benjamini–Hochberg method to correct for multiple testing. Using an FDR of <10%, we identified 29 transcripts whose pre-immunization expression levels correlated with age. This signature strongly clustered in a network centered around type I interferons (IFNα and IFNβ) and pro-inflammatory cytokines (IL-12, GM-CSF, and type II interferons TNFα and IFNγ) and was able to generally separate young and old adults’ pre-immunization following hierarchical cluster analysis and PCA (Figure 3). Applying an FC cutoff of >1.25 reduced the 29-gene signature to a 10-gene signature with improved accuracy (LOOCV of 70%) and discriminatory power (*p* = 0.031) (Figure S1 in Supplementary Material). Our finding that pro-inflammatory pathways predominate in elderly persons is in agreement with the study by Fourati et al., where genome-wide RNA expression profiles were compared between 30 participants aged 25–40 years and 144 participants aged ≥65 in Canada, which validates the results observed in our smaller-sized cohort (31).

To investigate whether pre-immunization gene expression profiles correlated with different responses to the hepatitis B vaccine, participants were categorized independently of age, but based on their anti-HBs concentrations 4 weeks after the third dose of the primary series (week 30) or 4 weeks after the booster vaccination. Individuals reaching anti-HBs concentrations of ≥10,000 IU/l were considered as high responders. Based on this criterion, five out of eight (62.5%) young adults were considered high responders during primary vaccination while the large majority of old adults [six out of seven (85.7%)] did not reach anti-HBs concentrations of ≥10,000 IU/l, suggesting a strong correlation between chronological age and lower vaccine responsiveness during primary vaccination (chi-squared test: *p* = 0.036). By contrast, 6 out of 12 (50.0%) young adults and 3 out of 8 (37.5%) old adults were considered high responders during booster vaccination (chi-squared test: *p* = 0.582), corroborating the observation that the increase in anti-HBs concentration was only significantly different between the age groups after primary vaccination, but not after booster vaccination (Figures 1 and 2).

**Figure 4:**
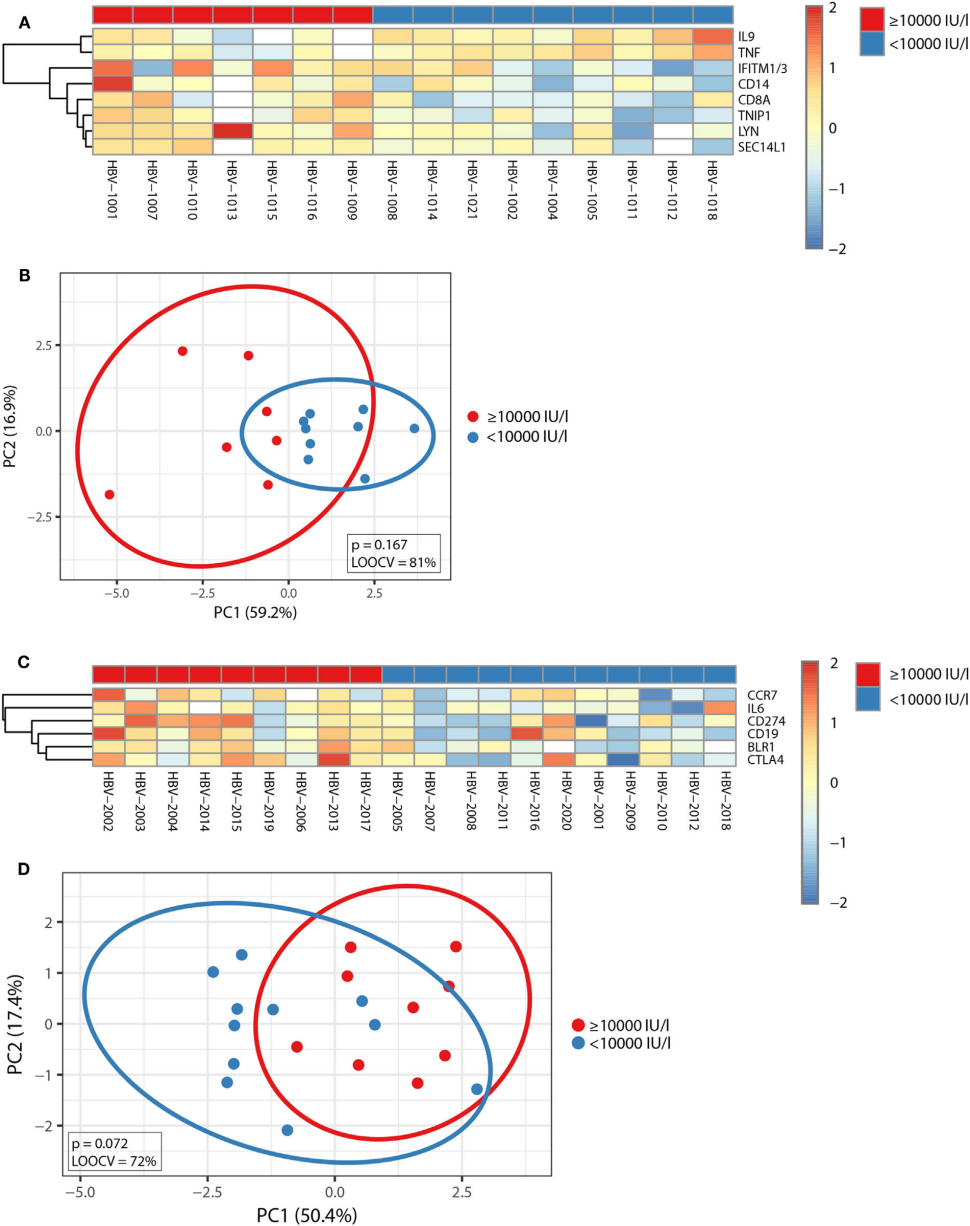
Correlation between pre-immunization transcriptomic profiles and vaccine responsiveness. **(A,C)** The median-centered gene expression of the transcripts whose pre-immunization expression levels correlated with vaccine responsiveness independent of age is represented using a blue to yellow to red color scale. Rows and columns correspond to the genes and the profiled samples, respectively. The vaccine responder groups are presented in colored squares above each sample. **(A)** It depicts the eight-gene signature identified after primary vaccination and **(C)** the six-gene signature identified after booster vaccination, respectively. **(B,D)** PCA analysis of the gene expression profile of the eight-gene signature identified during primary vaccination **(B)** and the six-gene signature identified during booster vaccination **(D)**. Blue and red spheres represent individuals with anti-HBs concentrations of <10,000 and ≥10,000 IU/l, respectively.

**Figure 5:**
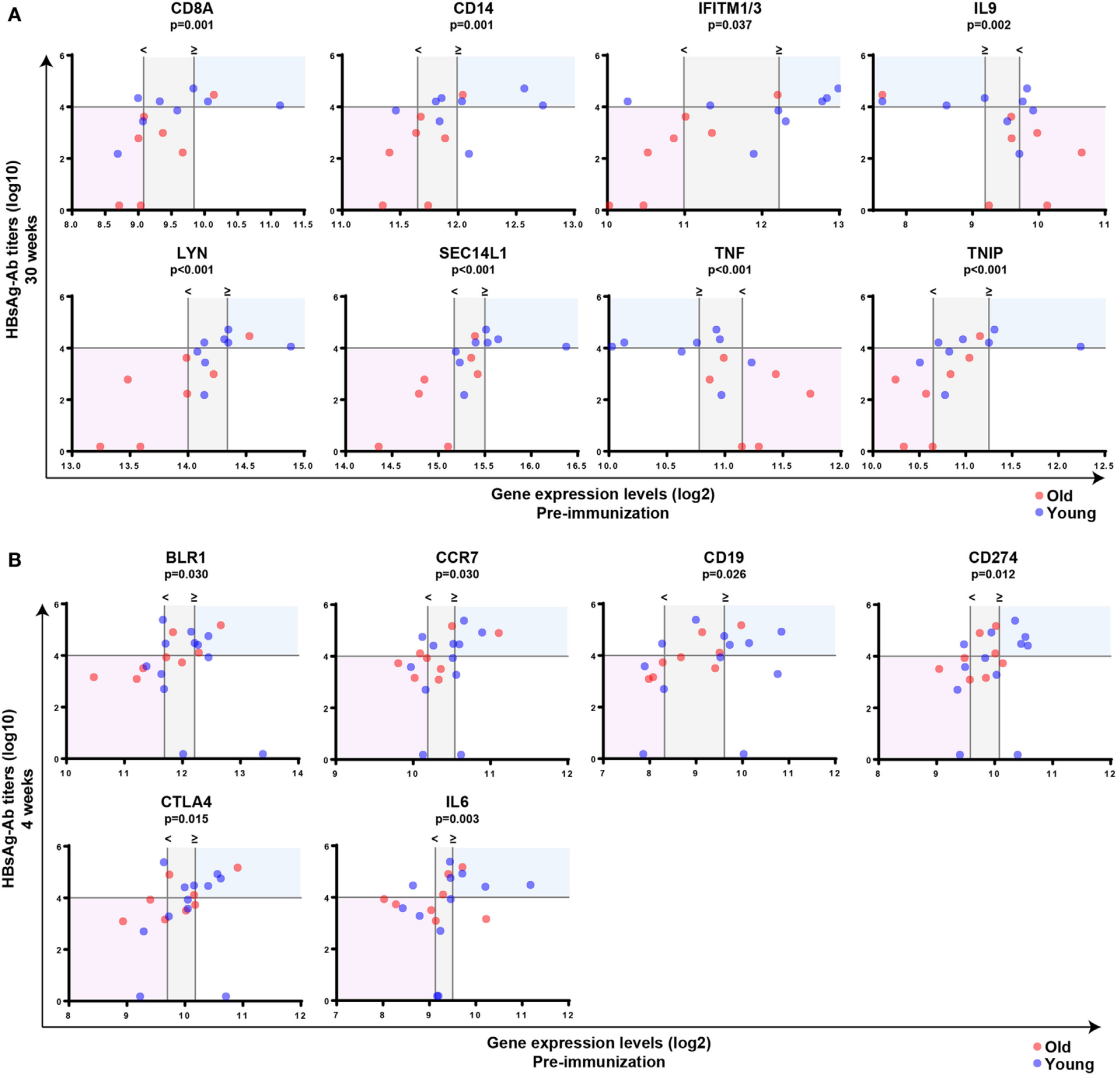
Correlation between pre-immunization expression levels of single genes and vaccine responsiveness. Correlation between pre-immunization expression levels of single genes (log2-transformed) and anti-HBs concentrations (log10-transformed) at week 30 following primary vaccination **(A)** or week 4 after booster vaccination **(B)** independent of age. Mann–Whitney *U*-test was used to identify transcripts differentially expressed between individuals with anti-HBs concentrationsof ≥10,000 or <10,000 IU/l independent of age, and *p*-values were adjusted using the Benjamini–Hochberg method to correct for multiple testing. The horizontal line indicates an anti-HBs concentration of 10,000 IU/l, which was used as a cutoff value to categorize participants. The vertical lines represent the median gene expression levels of the individuals with anti-HBs concentrations of <10,000 IU/l (<) and ≥10,000 IU/l (≥), and the area in between is indicated in gray. In case basal gene expression levels are significantly increased in high responders compared to persons with anti-HBs concentrations of <10,000 IU/l, the blue shading indicates the area above the median gene expression of the high responders and above an anti-HBs concentration of 10,000 IU/l. The area below the median gene expression levels of the persons with anti-HBs concentrations of <10,000 IU/l and below the anti-HBs concentration of 10,000 IU/l is shown in red. By contrast, if basal gene expression levels are significantly decreased in high responders compared to persons with anti-HBs concentrations of <10,000 IU/l, the opposite areas of the vertical lines representing the median gene expression levels are tinted using a similar color coding. Blue and red spheres represent young and old adults, respectively.

Subsequently, Mann–Whitney *U*-test was used to identify transcripts differentially expressed between individuals with anti-HBs concentrations below or above 10,000 IU/l independent of age, and *p*-values were adjusted with the Benjamini–Hochberg method to correct for multiple testing. Using an FDR of <10% and an FC of >1.25, we uncovered eight transcripts during primary vaccination and six transcripts during booster vaccination whose pre-immunization expression levels correlated with vaccine responsiveness (Figures 4A,C), and the identified gene signatures displayed an LOOCV accuracy of 81 and 72%, respectively (Figures 4B,D). In Figure 5, plots of the single genes encompassing the identified eight- and six-gene signatures are depicted. During primary vaccination, higher basal expression levels of CD8A, CD14, IFITM1/3, LYN, SEC14L1, and TNIP and lower basal transcriptomic levels of IL9 and TNF were associated with high anti-HBs concentrations (Figure 5A), while during booster vaccination, increased basal expression levels of BLR1, CCR7, CD19, CD274, CTLA4, and IL6 were correlated with higher anti-HBs concentrations (Figure 5B). Since BLR1, CCR7, CD19, CD274, and CTLA4 are all transcripts highly and preferentially expressed on T cells and/or B cells, these data suggest that during booster vaccination, vaccine responsiveness is mainly determined by differences in the representation and/or function of peripheral blood lymphocytes.

### Correlation Between the Kinetic Changes in Gene Expression Profiles and HBsAg-Specific Antibody Responses During Vaccination

**Figure 6:**
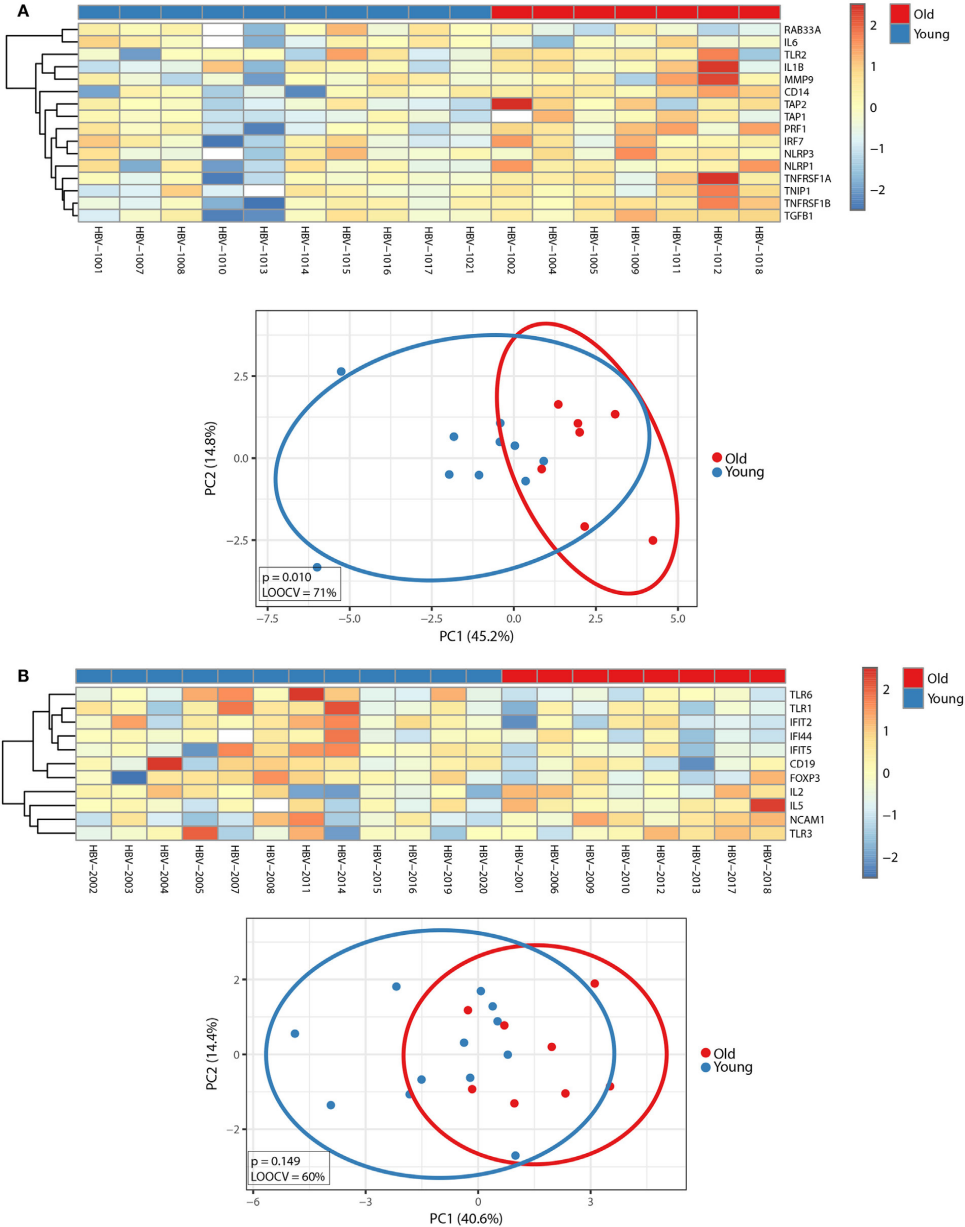
Impact of age on factorial changes (FCs) in transcriptomic profiles following primary or booster vaccination. FCs of transcriptomic profiles between d0 and d1 were calculated. Using an FDR of <10%, 16 and 11 transcripts were identified during the primary **(A)** and booster **(B)** vaccination, which correlated with age and displayed an FC of >1.25. (Top panels) The median-centered gene expression of differentially expressed genes is represented using a blue to yellow to red color scale. Rows and columns correspond to the genes and the profiled samples, respectively. The age groups are presented in colored squares above each sample. (Bottom panels) PCA analysis of the corresponding gene expression profiles. Blue and red spheres represent young and old adults, respectively.

Before exploring whether FCs in gene expression profiles before and after vaccination correlated with distinct responses to the hepatitis vaccine between young and old adults, we first investigated the impact of age on the FCs of the transcriptomic profiles between d0 and d1 during primary and booster vaccination. Using an FDR of <10%, we identified 16 and 11 transcripts during the primary and booster vaccination, respectively, that correlated with age and displayed an FC of >1.25 (Figure 6). While inflammasome markers, IFN-inducible genes, and genes encoding pro-inflammatory cytokines dominated the 16-gene signature that discriminated young and old adults during primary vaccination, the 11-gene signature that distinguished young and old adults during booster vaccination primarily encompassed genes coding pattern recognition receptors, immune cell subset markers as well as IFN-inducible genes. The capacity of the 16- and 11-gene signature to separate young and old adults is depicted by PCA analysis in Figure 6.

**Figure 7:**
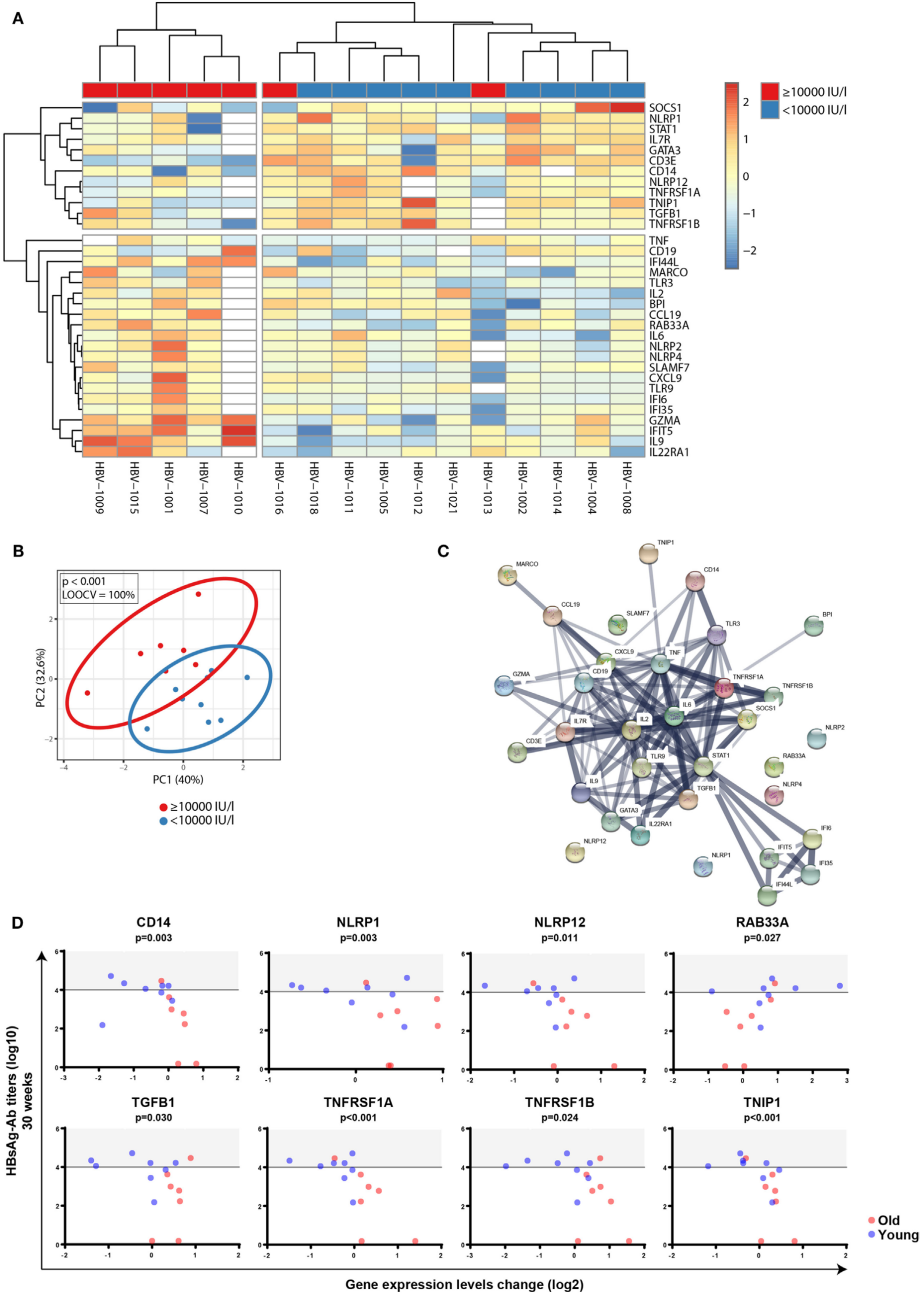
Correlation between factorial changes (FCs) of transcriptomic profiles following primary vaccination and vaccine responsiveness. FCs of transcriptomic profiles between d0 and d1 were calculated following primary vaccination independent of age. Mann–Whitney *U*-test was used to identify transcripts that were differentially regulated between individuals with anti-HBs concentrations of ≥10,000 and <10,000 IU/l independent of age, and *p*-values were adjusted with the Benjamini–Hochberg method to correct for multiple testing. Using an FDR of <10% and an FC of >1.25, 33 transcripts were found to be differentially expressed between the two groups. **(A)** The median-centered gene expression of the 33-gene signature is represented using a blue to yellow to red color scale. Rows and columns correspond to the genes and the profiled samples, respectively. The vaccine responder groups are presented in colored squares above each sample. **(B)** PCA analysis of the gene expression profile of the 33-gene signature. Blue and red spheres represent individuals with anti-HBs concentrations of <10,000 and ≥10,000 IU/l, respectively. **(C)** String network analysis of the proteins represented in the 33-gene signature. Individual proteins are displayed as nodes. Lines represent protein–protein interactions, and the thickness of the lines indicates confidence. **(D)** Plots of the eight single genes that showed both a correlation between FCs of transcriptomic expression levels before and after vaccination (d0 and d1) (log2-transformed) and anti-HBs concentrations at week 30 (log10-transformed) following primary vaccination (4 weeks after the third dose) as well as a correlation with age. The horizontal line indicates an anti-HBs concentration of 10,000 IU/l used as a cutoff value to categorize participants, and the area above the cutoff value (>10,000 IU/l) is highlighted in gray. Blue and red spheres represent young and old adults, respectively.

Next, to investigate whether FC in gene expression profiles before and after vaccination correlated with different responses to the hepatitis B vaccine, participants were again categorized into high responders (anti-HBs of ≥10,000 IU/l) and individuals not reaching these antibody levels, independent of age. FCs of transcriptomic profiles between d0 and d1 were calculated, and Mann–Whitney *U*-test was used to identify transcripts that were differentially regulated between the two groups independent of age. *P*-values were adjusted with the Benjamini–Hochberg method to correct for multiple testing. Using an FDR of <10% and an FC of >1.25, we uncovered 33 transcripts during primary vaccination that displayed an association with anti-HBs concentrations with excellent classifying capacity (Figures 7A,B). String network analysis revealed a network dominated by IFN-inducible genes, pro-inflammatory cytokines, inflammasome components, and immune cell subset markers (Figure 7C). In Figure 7D, plots of eight single genes that are part of the 33-gene signature are depicted, whose change in expression levels after vaccination not only correlated with anti-HBs concentrations after primary vaccination but also showed a significant correlation with chronological age (Mann–Whitney *U*-test: CD14  *p* < 0.001; NLRP1 *p* = 0.021; NLRP12 *p* = 0.042; RAB33A *p* = 0.044; TGFB1 *p* < 0.001; TNFRSF1A *p* < 0.001; TNFRSF1B *p* < 0.001; and TNIP1 *p* = 0.021). By contrast, none of the 144 transcripts displayed an association with anti-HB concentrations following booster vaccination. This finding is in concordance with the observation that the increase (fold-change) of anti-HBs concentrations after the booster vaccination (weeks 0–4) did not differ between high responders and individuals with anti-HBs of <10,000 IU/ml at week 4 (median 469.4-fold, range 72.5–743.8 versus median 467.1-fold, range 51.4–2325.6; *p* = 0.863, Mann–Whitney *U*-test). For this calculation, the two NRs were excluded.

## Discussion

In most cases, older adults are not immunologically naïve regarding the vaccines they receive. The most frequently studied vaccines in the elderly are against influenza and pneumococcal disease. Natural exposure to various influenza strains and pneumococcal serotypes induces immunological memory; therefore, even the first vaccination against those pathogens does not trigger a pure primary immune response. Primary, as well as booster immune responses against various pathogens, has been studied in the elderly (5–11), but a direct comparison using the same antigen is rarely done. We used HBsAg as a model antigen to investigate the impact of age on primary and booster immune responses following immunization in the absence of natural exposure.

Blood samples were taken at several time points after each dose of vaccine in order to evaluate not only the maximal response but also the development of antibody concentrations over time. Despite small sample sizes, which are a limitation of this study, we could observe a number of age-related differences in the response to primary hepatitis B vaccination. Firstly, the antibody response to primary vaccination was delayed in the older group compared to that in the younger adults. No antibody responses were detectable in the older vaccines after the first vaccination, and only 30% of the older participants showed an increase in antibodies after the second dose. By contrast, all but one young participant had mounted protective antibody levels 4 weeks after the second dose. In order to achieve protection, three doses of vaccine are needed for the elderly. This finding is of particular relevance when hepatitis B vaccine is administered as a travel vaccine, as usually only two doses of vaccine are administered before the travel activity. An age-associated delay in primary immune responses has also been described for yellow fever vaccine, with only 50% seroprotection in the older compared to 77% in the younger age group at day 10 after vaccination (32). Age has been reported to be a risk factor for being an NR to HBV vaccination in individual studies (33–36), as well as in meta-analyses (37, 38). In concordance with these studies, we observed NRs in the old, but not in the young age group. It has been shown previously that only 34% of an old and potentially frail (median age 72 years, range 57–95) cohort in a long-term care facility developed anti-HBs concentrations of >10 IU/ml after three doses of hepatitis B vaccination (39). The decline of antibody concentrations over time (weeks 30–52) was similar for both age groups.

By contrast, both young and older adults respond similarly to booster vaccination against HBV, and the decline of antibodies over 6 months did not show age-related differences. We have previously reported that recall responses to other vaccine antigens are lower in the elderly compared to younger adults (9, 11), but this was not the case in the current study. A prerequisite for successful booster vaccination is adequate priming. It can be speculated that primary vaccination (e.g., against tetanus and diphtheria), which is administered in early childhood, might not have been complete for all older adults contributing to low responses to booster vaccination in old age. All participants of the current study had completed a three-dose primary series at least 10 years prior to enrollment and at least for the older cohort, primary vaccination had been administered during adulthood. Studies indicate that immunological memory remains intact for at least 20 years among healthy vaccinated individuals who initiated hepatitis B vaccination >6 months of age (40). Even after the loss of anti-HBs, T cells may still confer protection (41). In a long-term follow-up of two clinical trials performed in the early 1990s, almost all participants still had detectable anti-HBs 20 years after primary vaccination (42). It has been reported that approximately 20% of health-care workers did not possess detectable HBsAg-specific antibodies more than 10 years after the primary vaccination. Booster vaccination led to an increase of antibody concentrations in almost all participants (43). Notably, all participants of these two studies received the primary vaccination series as adults. Primary vaccination of adolescents (12–15 years) resulted in 80% of the vaccines retaining specific antibodies 15 years later. Upon booster vaccination of 19 individuals without residual antibodies, all but one mounted a robust anamnestic response (44). By contrast, several studies showed that only 20–30% of adolescents who received primary vaccination in infancy still had protective anti-HBs levels around age 18 (45–47). The age when primary vaccination is received appears to play an important role in the long-term maintenance of anti-HBs. Among vaccinated cohorts who initiated hepatitis B vaccination at birth, long-term follow-up studies are ongoing to determine the duration of vaccine-induced immunity (40). Leuridan and Van Damme have summarized several studies from different countries in which booster vaccination was administered to individuals who had received primary vaccination in infancy, but had antibody concentrations below 10 IU/l several years later. Anamnestic responses to the booster vaccination were less frequent with increasing time since the primary vaccination and ranged from 100%, 5 years after the primary vaccination to 75.6% after 18–23 years (48). In our small cohort, two young participants had received primary vaccination in infancy more than 30 years ago. Both individuals did not have any residual antibodies and they did not mount sufficient antibody responses after the booster vaccination. No information was available on their anti-HBs responses after the primary vaccination. Therefore, we cannot rule out the possibility that the NRs never had sufficient antibody responses after primary vaccination, as it has been reported that approx. 5% of vaccinated children do not develop antibodies after primary vaccination (49). By contrast, the individuals with anti-HBs concentrations of <10 IU/ml before the booster, who had received primary vaccination as adults, responded adequately to the booster vaccination. It can be speculated that the rate of successful booster vaccination declines with increasing time since the primary vaccination and that age at the time of primary vaccination also plays a role. Further studies in larger cohorts would be necessary to determine optimal booster schedules, which take into account the age at the time of primary vaccination and the response to this primary series as well as residual antibodies and time since the last vaccination.

Latent infection with CMV has a substantial impact on the composition of the T cell pool (50–53), and an impact of CMV seropositivity on vaccine-induced antibody responses has been suggested (54–56), but was not confirmed in other studies (57, 58). CMV serostatus was determined for the participants of the current study, but the sample size was not sufficient to reliably determine the impact of CMV on primary and booster antibody responses in this cohort.

Because the molecular mechanisms underlying age-related hyporesponsiveness to vaccination remain unclear, whole blood samples for focused gene expression profiling were collected before (d0) and after (d1) receiving the first dose of primary vaccine or booster vaccine. Prior to vaccination, significant age-related changes were found that strongly clustered in a network centered around type I interferons and pro-inflammatory cytokines, and these pathways have been implicated in promoting immunosenescence in older adults (Figure 3) (59). Our observation that pro-inflammatory pathways prevail in the elderly is consistent with a larger study in Canada, where genome-wide RNA expression profiles were compared between young and old adults (30). Importantly, several studies have shown that naïve T cells decrease, while highly differentiated effector and memory T cells accumulate with chronological age (60, 61). This change is not only reflected by an alteration in cell numbers but also reflected by a change in the expression levels of transcripts that are highly and preferentially expressed in T and B lymphocytes (13, 14). In agreement with this view, expression levels of T cell and B cell subset markers (CD3E, IL7R, CD19, BLR1) were altered in old adults compared to younger participants, likely reflecting a shift in the relative abundance of peripheral blood lymphocytes with aging. Moreover, the expression of PTPRCv1 (CD45RA) located on naïve T cells was higher in younger participants, while the expression of PTPRCv2 (CD45RO) located on memory T cells was higher in older participants. This finding is consistent with a progressive increase of memory T cells with chronological age and may therefore reflect age-related changes in immune function.

A correlation between pre-immunization expression levels and vaccine responsiveness during primary and booster vaccination could only be established for a few genes (Figures 4 and 5). However, during booster vaccination, five of the six genes encompassing a six-gene signature encoded transcripts that were highly and preferentially expressed on T cells and/or B cells (BLR1, CCR7, CD19, CD274, and CTLA4), indicating that specifically during booster vaccination, vaccine responsiveness is largely determined by variances in the representation and/or function of naïve and memory B cell and T cell subsets.

In contrast to pre-immunization transcriptomic profiles, FCs in gene expression profiles before and after primary vaccination identified a larger 33-gene signature that correlated with different responses to the HBV vaccine (Figure 7), revealing a network dominated by IFN-inducible genes, pro-inflammatory cytokines, inflammasome components, and immune cell subset markers. Type I interferons have been shown to enhance the development of CD4 and CD8 central memory T cells as well as CD4 and CD8 effector memory T cells (62) and to improve B cell function by amplifying the B cell receptor signal (63), providing a plausible explanation for the observed positive association between genes induced by type I interferons and antibody titers. By contrast, a negative association between the induction of regulatory T cell-associated genes (CD3E, IL7R, TGFB1) and antibody titers was found, speculating that in individuals with antibody concentrations of <10,000 IU/l, the accelerated induction/recruitment of regulatory T cells compromises the magnitude and duration of inflammatory responses that are required for optimal antibody production (64). Another prominent group of genes identified in the network were inflammasome components. Reports about the role of these components during vaccination or B cell function are scarce, but it has been documented that the stimulation of NLRP3 by fungal antigens modulated IgM production (65). In addition, NLRP3^−/−^ mice demonstrated a decreased vaccine efficacy as measured by antibody production (66). Together, these data suggest that those signaling networks that change with progressive age, such as inflammation status, contribute to hyporesponsiveness in the elderly during primary vaccination. In contrast to primary vaccination, no correlation between FCs of transcriptomic profiles and vaccine responsiveness could be identified during booster vaccination. This finding is in concordance with the fact that the increase (fold-change) of anti-HBs concentrations after the booster vaccination did not differ between individuals with high and low antibody concentrations at week 4. Our results document that primary differs from booster vaccination in old age, regarding antibody responses as well as at the level of gene signatures.

## Data Availability

The raw data supporting the conclusions of this manuscript will be made available by the authors upon request.

## Ethics Statement

The protocol was approved by the ethics committee of the Innsbruck Medical University. All participants gave their written informed consent in accordance with the Declaration of Helsinki. This trial was registered at the EU Clinical Trial Register (EU-CTR) with the EUDRACT-Nr. 2013-002589-38.

## Author Contributions

BW, BG-L, MH, and TO designed the study. BW, MH, and TB performed experiments. BW, MH, and RP analyzed data. and BW, MH, TO, and BG-L wrote the manuscript. All authors contributed to manuscript revision, read, and approved the submitted version.

## Conflict of Interest Statement

The authors declare that the research was conducted in the absence of any commercial or financial relationships that could be construed as a potential conflict of interest.

## Footnotes

^1^http://biit.cs.ut.ee/clustvis/ (Accessed: November 26, 2017).

^2^https://string-db.org/ (Accessed: December 5, 2017).
