# Supplementary figures and images for: Impaired Immune Response to Primary but Not to Booster Vaccination Against Hepatitis B in Older Adults
Source: Front Immunol. 2018 May 15;9:1035. doi: 10.3389/fimmu.2018.01035 (PMC5962691; doi:10.3389/fimmu.2018.01035)

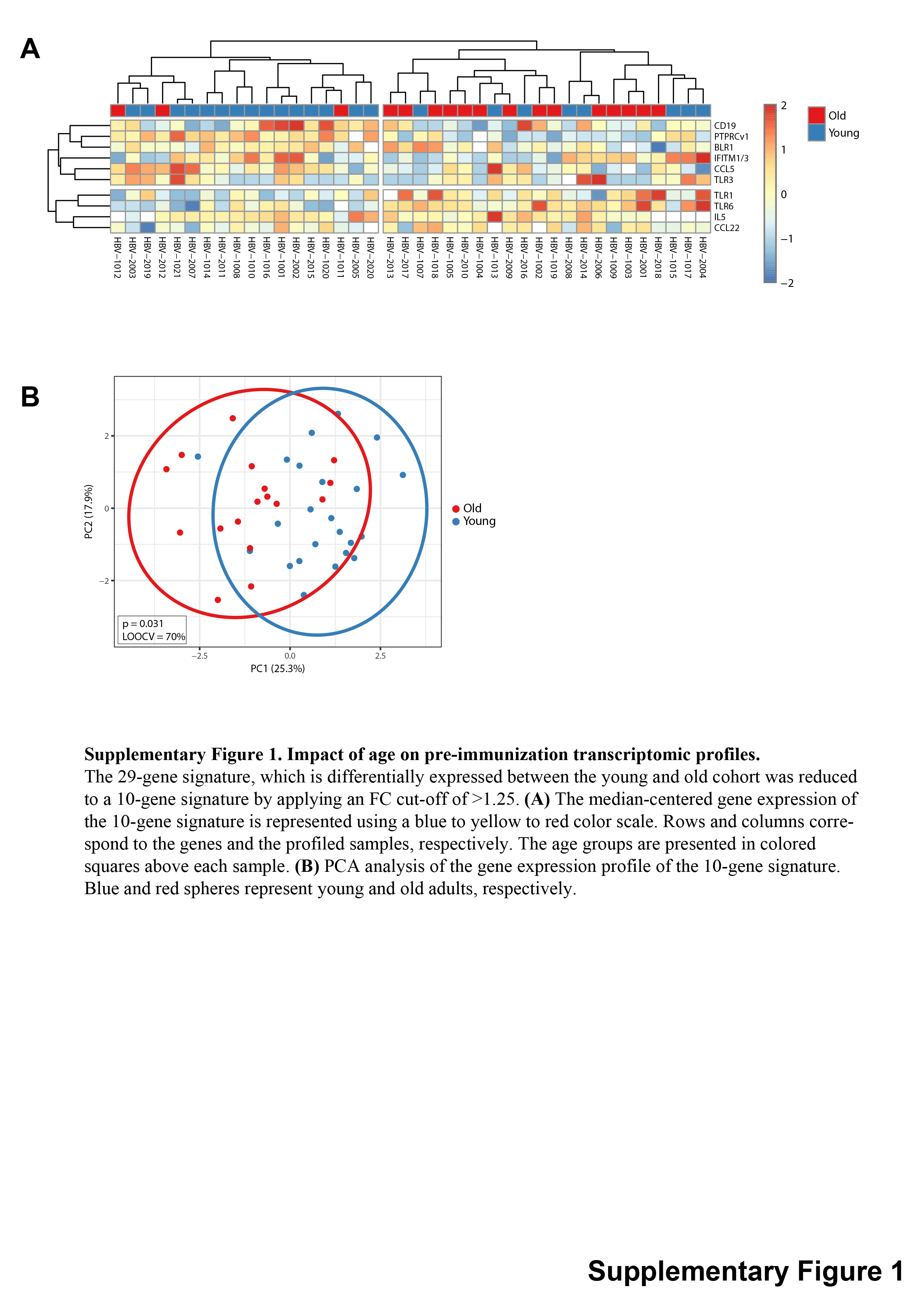

Supplement: Supplementary file 1 [file image_1.tif]
