# Supplementary material for: Impaired Immune Response to Primary but Not to Booster Vaccination Against Hepatitis B in Older Adults
Source: Front Immunol. 2018 May 15;9:1035. doi: 10.3389/fimmu.2018.01035 (PMC5962691; doi:10.3389/fimmu.2018.01035)
Supplement: Supplementary file 2 [file table_1.docx]

| **Supplementary Table 1. List of target genes for dcRT-MLPA.** | | | | | | |  |  | | | | |  | | | |  | |  | |
| --- | --- | --- | --- | --- | --- | --- | --- | --- | --- | --- | --- | --- | --- | --- | --- | --- | --- | --- | --- | --- |
|  | | |  |  | |  | | |  |  | | | |  | | | |  | |  |
| **Immune cell subset markers** |  | **Treg associated genes** | | |  | **IFN signaling genes** | | | | |  | **Chemokines** | | |  | **Cell Growth / Proliferation** | | | | |
| CD19 |  | CCL4 | | |  | CD274 | | | | |  | CCL11 | | |  | AREG | | | | |
| NCAM1 |  | CTLA4 | | |  | FCGR1A | | | | |  | CCL13 | | |  | BMP6 | | | | |
| **T cell subset markers** |  | FOXP3 | | |  | GBP1 | | | | |  | CCL19 | | |  | EGF | | | | |
| AIRE |  | IL2RA | | |  | GBP2 | | | | |  | CXCL9 | | |  | TGFBR2 | | | | |
| CCR7 |  | LAG3 | | |  | GBP5 | | | | |  | CX3CL1 | | |  | VEGF | | | | |
| CD3E |  | TGFB1 | | |  | IFI6 | | | | |  | **Pattern recognition receptors** | | |  | **Small GTPases/(Rho) GTPase activating proteins** | | | | |
| CD4 |  | TNFRSF18 | | |  | IFI16 | | | | |  | CD209 | | |  | ASAP1 | | | | |
| CD8A |  | **Cytotoxicity markers** | | |  | IFI35 | | | | |  | CLEC7A | | |  | RAB13 | | | | |
| IL7R |  | GNLY | | |  | IFI44 | | | | |  | MRC1 | | |  | RAB24 | | | | |
| PTPRCv1 |  | GZMA | | |  | IFI44L | | | | |  | MRC2 | | |  | RAB33A | | | | |
| PTPRCv2 |  | GZMB | | |  | IFIH1 | | | | |  | NOD1 | | |  | TAGAP | | | | |
| **Th1 associated genes** |  | PRF1 | | |  | IFIT2 | | | | |  | NOD2 | | |  | TBC1D7 | | | | |
| CXCL10 |  | **Apoptosis/survival** | | |  | IFIT3 | | | | |  | TLR1 | | |  | **Anti-microbial activity** | | | | |
| IFNG |  | CASP8 | | |  | IFIT5 | | | | |  | TLR2 | | |  | BPI | | | | |
| IL1B |  | BCL2 | | |  | IFITM1/3 | | | | |  | TLR3 | | |  | LTF | | | | |
| IL2 |  | FASLG | | |  | INDO | | | | |  | TLR4 | | |  | **Mitochondrial stress / Proteasome** | | | | |
| IL15 |  | FLCN1 | | |  | IRF7 | | | | |  | TLR5 | | |  | HPRT | | | | |
| TBX21 |  | TNFRSF1A | | |  | OAS1 | | | | |  | TLR6 | | |  | **E3 ubiquitin protein ligases** | | | | |
| TNF |  | TNFRSF1B | | |  | OAS2 | | | | |  | TLR7 | | |  | NEDD4L | | | | |
| **Th2 associated genes** |  | **Myeloid associated genes** | | |  | OAS3 | | | | |  | TLR8 | | |  | **Scavenger receptors** | | | | |
| GATA3 |  | CCL2 | | |  | SOCS1 | | | | |  | TLR9 | | |  | MARCO | | | | |
| IL4 |  | CCL3 | | |  | STAT1 | | | | |  | TLR10 | | |  | **Transcriptional regulators/activators** | | | | |
| IL4δ2 |  | CCL5 | | |  | STAT2 | | | | |  | **Inflammasome components** | | |  | CAMTA1 | | | | |
| IL5 |  | CCL22 | | |  | TAP1 | | | | |  | NLRC4 | | |  | TWIST1 | | | | |
| IL6 |  | CD14 | | |  | TAP2 | | | | |  | NLRP1 | | |  | ZNF331 | | | | |
| IL10 |  | CD163 | | |  | **Inflammation** | | | | |  | NLRP2 | | |  | ZNF532 | | | | |
| IL13 |  | CXCL13 | | |  | DSE | | | | |  | NLRP3 | | |  | **G protein-coupled receptors** | | | | |
| **Th9 associated genes** |  | IL12A | | |  | MMP9 | | | | |  | NLRP4 | | |  | BLR1 | | | | |
| IL9 |  | IL12B | | |  | SPP1 | | | | |  | NLRP6 | | |  | **Reference genes** | | | | |
| **Th17 associated genes** |  | IL23A | | |  | TIMP2 | | | | |  | NLRP7 | | |  | ABR | | | | |
| IL17A |  | FPR1 | | |  | TNIP1 | | | | |  | NLRP10 | | |  | B2M | | | | |
| IL22RA1 |  | **Cell activation** | | |  | **Intracellular transport** | | | | |  | NLRP11 | | |  | GAPDH | | | | |
| RORC |  | HCK | | |  | KIF1B | | | | |  | NLRP12 | | |  | GUSB | | | | |
|  |  | LYN | | |  | SEC14L1 | | | | |  | NLRP13 | | |  |  | | | | |
|  |  | SLAMF7 | | |  |  | | | | |  |  | | |  |  | | | | |
